# iMIL4PATH: A Semi-Supervised Interpretable Approach for Colorectal Whole-Slide Images

**DOI:** 10.3390/cancers14102489

**Published:** 2022-05-18

**Authors:** Pedro C. Neto, Sara P. Oliveira, Diana Montezuma, João Fraga, Ana Monteiro, Liliana Ribeiro, Sofia Gonçalves, Isabel M. Pinto, Jaime S. Cardoso

**Affiliations:** 1 Institute for Systems and Computer Engineering, Technology and Science (INESC TEC), 4200-465 Porto, Portugal; sara.i.oliveira@inesctec.pt (S.P.O.); jaime.cardoso@inesctec.pt (J.S.C.); 2 Faculty of Engineering, University of Porto (FEUP), 4200-465 Porto, Portugal; 3 IMP Diagnostics, 4150-146 Porto, Portugal; diana.felizardo@impdiagnostics.com (D.M.); ana.monteiro@impdiagnostics.com (A.M.); liliana.ribeiro@impdiagnostics.com (L.R.); sofia.goncalves@impdiagnostics.com (S.G.); isabel.macedo.pinto@impdiagnostics.com (I.M.P.); 4 School of Medicine and Biomedical Sciences, University of Porto (ICBAS), 4050-313 Porto, Portugal; 5 Cancer Biology and Epigenetics Group, IPO-Porto, 4200-072 Porto, Portugal; 6 Department of Pathology, IPO-Porto, 4200-072 Porto, Portugal; joao.meneses.fraga@ipoporto.min-saude.pt

**Keywords:** weakly supervised learning, semi-supervised learning, multiple-instance learning, interpretability, computational pathology, colorectal cancer

## Abstract

**Simple Summary:**

Nowadays, colorectal cancer is the third most incident cancer worldwide and, although it can be detected by imaging techniques, diagnosis is always based on biopsy samples. This assessment includes neoplasia grading, a subjective yet important task for pathologists. With the growing availability of digital slides, the development of robust and high-performance computer vision algorithms can help to tackle such a task. In this work, we propose an approach to automatically detect and grade lesions in colorectal biopsies with high sensitivity. The presented model attempts to support slide decision reasoning in terms of the spatial distribution of lesions, focusing the pathologist’s attention on key areas. Thus, it can be integrated into clinical practice as a second opinion or as a flag for details that may have been missed at first glance.

**Abstract:**

Colorectal cancer (CRC) diagnosis is based on samples obtained from biopsies, assessed in pathology laboratories. Due to population growth and ageing, as well as better screening programs, the CRC incidence rate has been increasing, leading to a higher workload for pathologists. In this sense, the application of AI for automatic CRC diagnosis, particularly on whole-slide images (WSI), is of utmost relevance, in order to assist professionals in case triage and case review. In this work, we propose an interpretable semi-supervised approach to detect lesions in colorectal biopsies with high sensitivity, based on multiple-instance learning and feature aggregation methods. The model was developed on an extended version of the recent, publicly available CRC dataset (the CRC+ dataset with 4433 WSI), using 3424 slides for training and 1009 slides for evaluation. The proposed method attained 90.19% classification ACC, 98.8% sensitivity, 85.7% specificity, and a quadratic weighted kappa of 0.888 at slide-based evaluation. Its generalisation capabilities are also studied on two publicly available external datasets.

## 1. Introduction

Nowadays, colorectal cancer (CRC) is the third most incident (11% of all cancers) and the second most deadly type of cancer worldwide (with a 9.5% mortality rate, only surpassed by lung cancer, with 18.2%), according to the Globocan estimated data for 2020 [1]. In addition, the development of this type of cancer is greatly influenced by multiple factors, such as lifestyle, genetics, and environmental factors. Thus, with the world becoming richer, and people adopting a western lifestyle, the incidence rates of CRC are expected to increase [2,3]. However, despite the statistics, CRC is preventable and curable if detected in its earlier stages by effective screening through medical examination, imaging techniques and colonoscopy [4,5].

While CRC can be detected by imaging techniques, further diagnosis is always based on samples obtained from biopsies and assessed by pathologists. Regarding the neoplasia development stage, these samples can be stratified in non-neoplastic (NNeo), low-grade dysplasia (LGD), high-grade dysplasia (HGD, including intramucosal carcinomas) and invasive carcinomas, from the lowest to the highest level of cancer progression, respectively. Although this grading is somewhat subjective [6], the most recent guidelines from the European Society of Gastrointestinal Endoscopy (ESGE), as well as those from the US multi-society task force on CRC, continue to recommend surveillance for polyps with high-grade dysplasia, regardless of their size [5,7]. Thus, this remains an important task for pathologists when assessing colorectal tissue samples.

Digitised slides are becoming increasingly available, in the form of whole-slide images (WSI), with more laboratories adopting a digital workflow [8,9,10]. Although it requires an additional scanning step, WSI allows pathologists to easily access old cases, share data and peer-review cases more quickly [11,12]. Moreover, digital pathology has created many research opportunities in the computer vision field, with the high dimensions of WSI and the complex nature of pathology assessment bringing new challenges to advanced automatic image processing systems [13,14,15,16]. Thus, the development of robust and high-performance algorithms, which are transparent and as interpretable as possible, can be valuable to assist pathologists in their daily workload [11,12]. However, despite its clinical relevance, the application of AI for CRC diagnosis from WSI is still poorly explored and there are some limitations that hinder its application in clinical practice, as recently highlighted by Oliveira et al. [17].

Currently, most of the works published on CRC diagnosis focus on classifying cropped regions of interest, or even small tiles, rather than the much more challenging task of assessing the entire WSI, as noted in recent reviews [17,18,19,20]. Nevertheless, some authors have already presented approaches based on the evaluation of the entire slide of colorectal samples. Iizuka et al. [21] proposed the combination of an Inception-v3 network (tile classifier) with a recurrent neural network (RNN), as a tile aggregator, to classify H&E colorectal WSI into non-neoplastic, adenoma (AD) and adenocarcinoma (ADC), obtaining an area under the curve (AUC) of 0.962 and 0.992 for colorectal AD and ADC, respectively. This tiling scheme is usual in computational pathology, since WSI have high dimensions, usually over 50,000 × 50,000 pixels, due to their pyramidal format (with several magnification levels) [22]. Thus, these images need to be decomposed into smaller tiles to fit into the memory of the graphics processing units (GPU) often used to train deep learning (DL) models. Wei et al. [23] aimed to distinguish different types of CRC adenomas in H&E stained slides. The model is an ensemble of the five versions of the ResNet architecture for classifying tiles and a hierarchical classifier for predicting a slide diagnosis, reaching an accuracy (ACC) of 93.5% on the internal test set, and 87.0% on the external test set. Song et al. [24] presented an approach based on a modified DeepLab-v2 network for tile classification and pixel probability thresholding to detect CRC adenomas, which achieved an AUC of 0.92 and an ACC of over 90% on an independent test set. Similarly, Xu et al. [25] proposed an Inception-v3-based model as a tile classifier, and a final slide classification based on tile prediction probability for detecting CRC, obtaining an ACC of 99.9% for normal slides and 93.6% for cancer slides. In addition, using the Inception-v3 architecture, Wang et al. [26,27] developed a framework to detect tumours which retrieves the final classification of a slide and also a map of tumour regions. From the tile classifier, which distinguishes normal and cancer tiles, slide prediction is obtained with a tile-cluster-based aggregation: a WSI is positive if several positive patches are topologically connected as a cluster, e.g., four patches as a square, and negative otherwise. This approach was tested on several WSI sets, achieving accuracies higher than 93%, an AUC higher than 91%, sensitivities higher than 92% and specificities higher than 88%. Marini et al. [28] proposed a multi-scale task multiple instance learning (MuSTMIL) method to classify five colon-tissue findings: normal glands, hyperplastic polyps, low-grade displasias, high-grade displasias and carcinomas. Using multiple scale branches, in a multi-task network, the model combines features from several magnification levels of a slide in a global prediction. This method reached an ACC of 87.0% and a 0.893 F1-score, in the binary setup, and an ACC of 85.7% and 0.682 F1-score, in the multi-class setup. More recently, Ho et al. [29] presented an algorithm that simultaneously segments glands, detects tumour areas and sorts the slides into low-risk (benign, inflammation or reactive changes) and high-risk (adenocarcinoma or dysplasia) categories. The authors proposed a Faster-RCNN architecture, with a ResNet-101 backbone network, for glandular segmentation of tiles, followed by a gradient-boosted decision tree for slide classification, using features such as the total area classified as adenocarcinoma or dysplasia, and the average prediction certainty for these areas. The model achieved an ACC of 79.3% with an AUC of 0.917, a sensitivity of 97.4% and a specificity of 60.3%.

The main goal of this work was to develop a system that is one step closer to being used by pathologists in their daily routine, which includes the following contributions: (1) an improved method that combines weakly and supervised learning methods to construct a novel system to diagnose CRC from digitised Haematoxylin-Eosin (H&E) stained slides, with high ACC and sensitivity; (2) a thorough comparison of several aggregation methods to increase the number of tiles used for predictions, which can reduce the number of false positives; (3) extensive experiments on an extended version of the publicly available CRC dataset; (4) a study of the model’s interpretability and capability to self-explain the diagnosis areas through the reconstruction of the slide with individual tile predictions without requiring added training. This latter contribution can be especially useful to guide pathologists’ attention towards the most relevant tissue areas within each WSI; and (5) evaluation of domain generalisation on two public colorectal WSI datasets (samples from the TCGA [30,31,32] repository and from the PAIP colorectal cohort [33]).

Besides this introduction, this paper consists of two more sections, followed by the conclusion, in Section 5. Section 2 includes the proposed methodology and a description of the datasets used. Then, the details and results of the conducted experiments are presented and discussed in Section 3.

## 2. Materials and Methods

### 2.1. Data Pre-Processing

**Figure 1:**
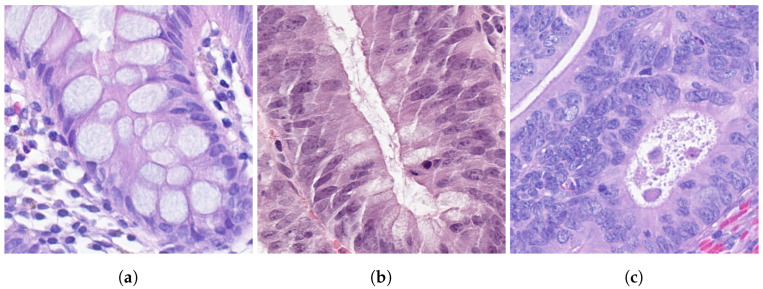
Examples of tiles with 512×512 pixels (40× magnification), representing each class: non-neoplastic (**a**), low-grade dysplasia (**b**) and high-grade dysplasia (**c**).

The H&E slide pre-processing includes an automatic tissue segmentation with Otsu’s thresholding [34] on the saturation (S) channel of the HSV colour space, obtaining the tissue regions clearly separated from the whitish background. This step, performed on the 32× downsampled slide, returned the mask used for tile extraction. Tiles with size of 512×512 pixels (Figure 1) were then extracted from the slide with original dimensions (without downsampling) at the maximum magnification (40×), provided they were completely within Otsu’s mask (100% tissue threshold). The tile size was chosen by empirical experiments, which showed that 512×512 is the best trade off between memory and performance. Larger sizes represent more context and tissue per tile, at the expense of memory and computation time. The threshold of 100% reduces the number of tiles by not including the tissue at the edges, which drastically decreases the computational cost, without hurting the performance of the model. Since the original size of each WSI and the amount of tissue per slide varies greatly, the number of tiles extracted also varies a lot.

### 2.2. Problem Definition

Automated diagnosis of colorectal cancer histological samples requires the use of images with large dimensions. In addition, the labelling of these images is difficult, expensive, and tedious. Therefore, the availability of WSIs is limited, and, when available, they often lack meaningful labelling: while slide-level diagnoses are generally available, detailed spatial annotations are almost always lacking. A prototypical example is the CRC dataset [17], containing 1133 colorectal H&E samples with slide-level diagnoses.

**Figure 2:**
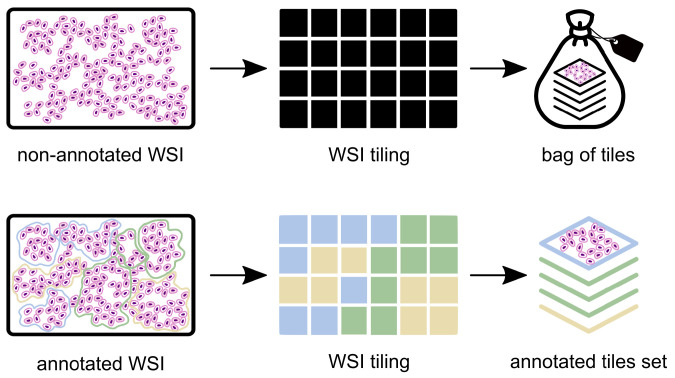
Labelling scheme: weakly annotated slides (**above**) have only a global label, from the pathology report, whereas a strongly annotated slide (**below**) has labels for each individual tile, retrieved directly from the pathologists’ spatial annotations.

Thus, following previous work on CRC diagnosis, and on automatic diagnostic systems in general, we assumed a semi-supervised learning procedure. A slide S can be viewed as a set of tiles $T_{s,n}$, where *s* is the index of the slide and $n\in \{1,\cdots ,n_{s}\}$ is the tile number. We assumed that there were individual labels $C_{s,n}\in \left\{C^{\left(1\right)},\cdots ,C^{\left(K\right)}\right\}$ for the tiles within the slide. The classes $C^{\left(k\right)}$ were considered ordered and correspond to the different diagnostic grades. For a strongly annotated slide, each corresponding tile label $C_{s,n}$ is known. In a weakly annotated slide, there is no access to those labels and they remain unknown during training. A weakly annotated slide has only a single label for the entire set (bag) of tiles, see Figure 2. Finally, we assumed that the slide label $C_{s}$ is the worst-case of the tile labels:

$$C_{s}=\max_{n}\left\{C_{s,n}\right\}.$$

If there is one tile in the set of tiles extracted from a slide that is classified as high-grade dysplasia, then the slide label will be the same. Second, if there is no dysplasia in any of the tiles, then the slide label is non-neoplastic. This learning setting corresponds to a simple generalisation of multiple-instance learning (MIL), from the binary problem to the ordinal classification problem.

### 2.3. Model Architecture

**Figure 3:**
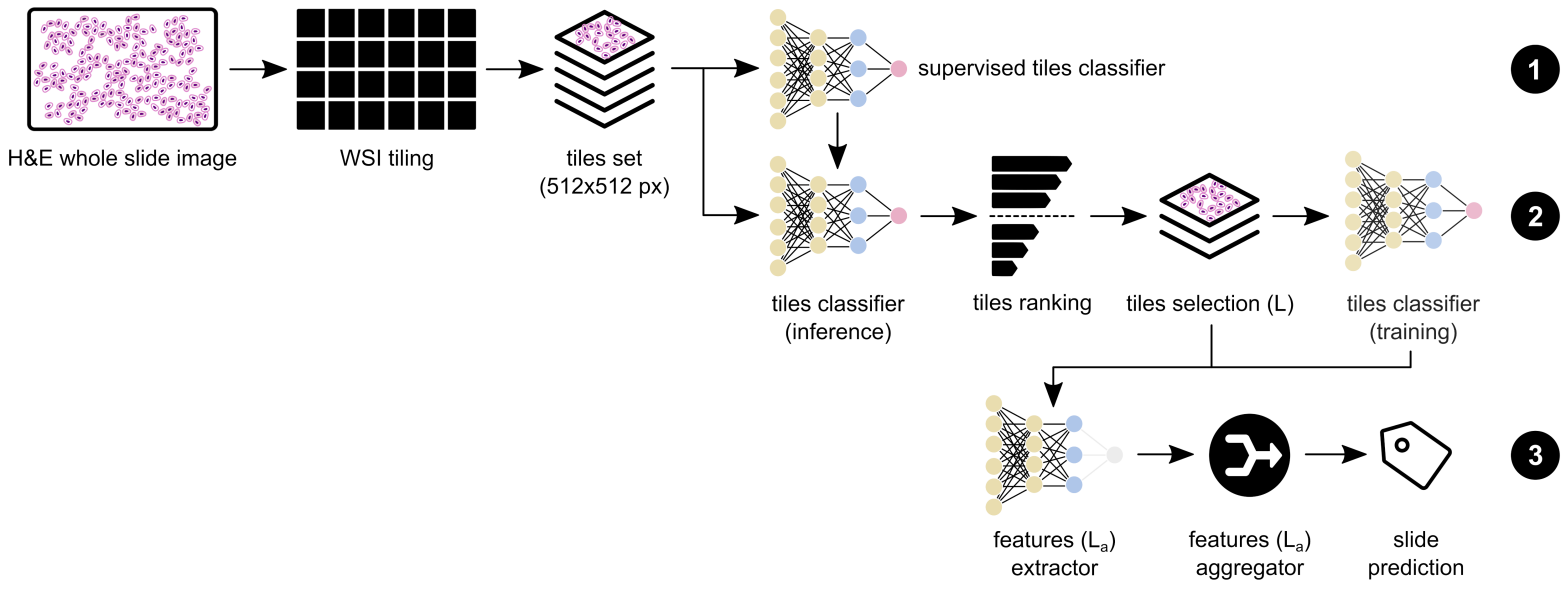
Proposed workflow for colorectal cancer diagnosis on whole-slide images, as a three-step method: (1) supervised tile classifier, using the annotated slides set; (2) weakly supervised tile classifier (initialised with the supervised weights), selecting the most relevant tiles by ranking by the expected values; and (3) whole-slide prediction by aggregating the features (obtained with the previous CNN model) extracted from the most relevant tiles.

With the CRC dataset, Oliveira et al. [17] presented an approach that has already introduced some modifications to the MIL method presented by Campanella et al. [14]. We further extended those modifications and adjusted them to better fit the requirements of an automatic CRC diagnosis system. In Figure 3, the architecture of our system is introduced, which is mainly composed of a supervised pre-training phase, to leverage the samples that include annotations (≈9% in the adopted dataset), a weakly supervised training phase, where all the dataset is used, and a final stage that includes feature extraction and training of an aggregation method. While supervised learning requires extensive use of annotations, we followed an approach that merges weakly and supervised learning, and uses less than one annotated sample per ten non-samples, while performing on par with the state-of-the-art methods.

#### 2.3.1. Supervised Pre-Training

The supervised training phase leverages the annotations of all tiles in the strongly annotated WSIs to train a ResNet-34 [35], which classifies into the three diagnostic classes by minimising a loss function based on the quadratic weighted kappa (QWK). The QWK loss is appropriate for ordinal data because it weights misclassifications differently, according to the equation:

(1) $$\kappa =1-\frac{\sum_{i,j=1}^{n}w_{ij}x_{ij}}{\sum_{i,j=1}^{n}w_{ij}m_{ij}}$$

where *K* is the number of classes, $w_{ij}$ belongs to the weight matrix, $x_{ij}$ belongs to the observed matrix and $m_{ij}$ are elements in the expected matrices. The n×n matrix of weights *w* is computed based on the difference between the actual and predicted class, as follows:

(2) $$w_{i,j}=\frac{{\left(i-j\right)}^{2}}{{\left(n-1\right)}^{2}}$$

As shown by Oliveira et al. [17], pre-training on a small set of data with supervised learning leads to faster convergence and also better results on all metrics.

During our studies, we found that the approach followed by Oliveira et al. [17], used as baseline, could be improved with increased pre-training. Compared to the weakly supervised training phase, the supervised training was significantly faster to complete an epoch. In addition, thus, with a trivial computational cost, it was possible to increase the number of pre-training epochs from two to five. This change positively impacted the algorithm’s performance on test set.

#### 2.3.2. Weakly Supervised Training

The weakly supervised training phase uses all the available training slides and only slide-level labels to complete the training of the deep network. The model is used to infer all tiles classes and then, based on those predictions, the tiles are ranked. We followed the approach of Oliveira et al. [17], which performed a tile ranking based on the expected value of the predictions.

For tile $T_{s,n}$, the expected value of the score is defined as

(3) $$E\left({\hat{C}}_{s,n}\right)=\sum_{i=1}^{K}i\times p\left({\hat{C}}_{s,n}=C^{\left(i\right)}\right)$$

where ${\hat{C}}_{s,n}$ is a random variable on the set of possible class labels $\left\{C^{\left(1\right)},\cdots ,C^{\left(K\right)}\right\}$ and $p\left({\hat{C}}_{s,n}=C^{\left(i\right)}\right)$ are the *K* output values of the neural network.

Despite the ranking of all tiles, only the worst tile (from a clinical point of view), i.e., the one with the highest expected value, was used to optimise the weights of the network. From the perspective of MIL, this corresponds to an aggregation function based on the maximum of the observations of the bag. This can slow down the training and even make it more unstable, especially in the first epochs, when the tile predictions are still very noisy. Therefore, instead of using only the tile with the highest expected value, we considered the generalisation of max function, $top_{L}\left(.\right)$, which keeps the first *L* tiles with the highest score. By changing the number of tiles used to optimise the network, we also increased the variability and possible changes between epochs. For example, it is more likely that none of the selected tiles will change if only one is selected. However, by selecting L>1, we increased the probability that the tiles will change in successive epochs while maintaining the stability of the training. Similar to the previous change, this one also resulted in a more robust model compared to the baseline.

After the model’s performance with the one tile MIL aggregation (L=1), and also after an in-depth analysis with pathologists, we decided that the WSI on the adopted dataset contained, on average, enough information to use at least five tiles. The definition of sufficient information was determined by the number of tiles that contained information related to the slide diagnosis. For instance, if a WSI label was from a high-grade dysplasia, only tiles with information of a possible high-grade dysplasia were considered to be useful, and, thus, tiles with only normal tissue should not be used to optimise the network weights. The value of *L* was then set to L=5, since this value represents a significant increase from L=1 and it does not use (in the majority of the slides) tiles with a potentially distinct diagnosis from the slide diagnosis.

There is growing concern regarding semi-supervised methods’ overconfident behaviour. There have also been works that aimed at addressing this problem on other tasks through network calibration [36]. However, in this specific scenario, it is believed that an over-confident model on the severe cases leads to fewer false negatives and higher sensitivity. In addition, thus, it is not seen as a potential problem of the model. On the other hand, the proposed aggregation approaches in the following section show properties that mitigate the risk of overconfidence.

#### 2.3.3. Feature Extraction and Aggregation

Regarding the max-pooling aggregation on multiple-instance learning approaches, one can argue about its robustness, such as the discussion presented, for example, by Campanella et al. [14], since it is a biased aggregation towards positive labels, and one small change can impact the entire tile classification. Hence, we studied the incorporation of shallow aggregation structures into our model to improve the results given by max-pooling.

It was found that the use of only one tile leads to a bias of the network towards more aggressive predictions. For this reason, we followed a strategy that has been adopted in other domains: the CNN was trained end-to-end as a classification model (using a combination of supervised pre-training and weakly supervised learning) and, after training, the fully-connected layer was removed. The network then output a feature vector for each tile, which were aggregated and used to train a supervised method at the slide-level to improve the classification capabilities of the system. For this problem, we chose to use $L_{a}$ feature vectors, corresponding to the $L_{a}$ tiles with the highest expected value for the score (Equation (3)). In our experimental study, $L_{a}$ was empirically set to 7, which represents a good trade off between additional information and the introduction of noise.

In order to compare different classifiers, we selected six aggregation models to test in our system:

- A support-vector machine (SVM) with a radial basis function kernel and a C of 1.0;
- A K-nearest neighbour (KNN) with a K equal to 5;
- A random forest (RF) with a max. depth of 4 and the Gini criterion;
- AdaBoost and XGBoost with 3000 and 5000 estimators, respectively;
- Two distinct multi-layer perceptrons (MLP) with two layers; the first MLP with layers of 75 and 5 nodes—MLP(75;5)—and a second one with layers of 300 and 50 nodes—MLP(300;50).

Besides these individual models, we also combined the previous ones into voting schemes, following a soft voting technique based on the probabilities of each model: SVM and KNN; and SVM, RF and KNN.

#### 2.3.4. Interpretability Assessment

Nowadays, deep learning models are becoming more complex and opaque. This is alarming, especially when we look at the potential applications of these models in the medical domain. If they are designed to work all by themselves, we need to ensure they are completely transparent. In addition, if they are to be used as a tool to help pathologists make a particular diagnosis and improve their confidence, then they must at least direct their focus to the areas relevant to the diagnosis.

It is necessary to understand the behaviour of the model in order to extend the validity of the typical analysis supported in metrics such as ACC, QWK and sensitivity. Therefore, a method was developed to generate visual explanations of model predictions. This method was constructed with the following ideas in mind: (a) for large WSI images, it is helpful to direct the pathologist to specific areas of high relevance; (b) since the model was not trained on tile ACC, it is sufficient if it is able to highlight a subset of the relevant tiles in a given area of interest; and (c) since, for the slide prediction, the model requires the processing of all tiles, creating a map of tile predictions does not require additional computational cost or idle time for the pathologist.

Given these ideas, the proposed method leverages the evaluation of the MIL method, which processes every tile in the WSI. Even if the tile is not selected for aggregation, it will be processed by the backbone network, which results in a tile-score prediction (c). These tile-level predictions are converted into colours based on the result of the Argmax function applied to their scores. Afterwards, these colours can be spatially allocated based on a remapping strategy from the tile at the original slide magnification to a 32× reduced WSI (a). In addition, while some of the predictions might be misclassified, the entire reconstruction of the WSI shall be sufficient to redirect the attention of pathologist towards the areas of interest (b).

### 2.4. Datasets

**Figure 4:**
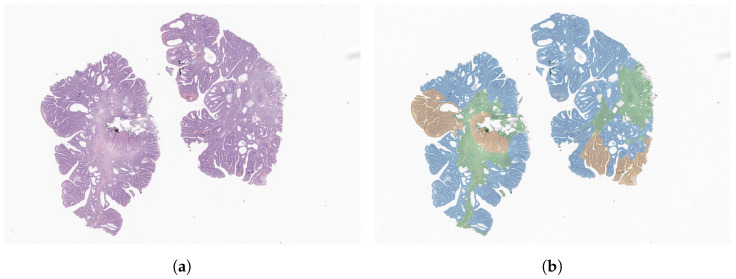
Example of a WSI (**a**), with manual segmentations overlayed (**b**). Tissue regions are annotated as non-neoplastic (green), low-grade (blue) or high-grade (yellow).

One of the datasets used in this work consists of 1133 colorectal biopsy and polypectomy slides (example in Figure 4a), from the recent and publicly available CRC dataset [17]. All samples are labelled within three categories: non-neoplastic (NNeo), low-grade lesions (LG), and high-grade lesions (HG).

The NNeo category includes normal colorectal mucosa, non-specific inflammation and hyperplasia. LG lesions correspond to conventional adenomas with low-grade dysplasia. HG lesions consist of conventional adenomas with high-grade dysplasia (including intra-mucosal carcinomas) and invasive adenocarcinomas. The dataset does not include either slides with suspicion of/known history of inflammatory bowel disease/infection, or any serrated lesions or other polyp types.

All slides were retrieved from the lab archive and digitised with Leica GT450 WSI scanners, at 40× magnification. Then, the slides were assessed by one of two pathologists and, when the diagnosis was different from the initial report, the case was rechecked and decided between the two. From the entire set, a small number of slides (*n* = 100) were also manually annotated (like the example in Figure 4b) by one of the pathologists and then rechecked by the other, using the Sedeen Viewer software [37]. For complex cases, or when an agreement could not be reached, a third pathologist reevaluated both the label and/or annotation.

This work also evaluated the proposed methodology with a second dataset (CRC+), which is an extended version of the one proposed by Oliveira et al. [17], with approximately 4× more samples (4433 colorectal H&E slides), of which a subset (*n* = 400) is annotated according to the guidelines followed on the original one [17,38]. The CRC dataset was still used for the selection and comparison of aggregation methods. The CRC+ was used to create a more robust test set and a larger training set to train the methods previously selected. Table 1 summarises the class distribution of annotated and non-annotated data, including the number of tiles obtained after the pre-processing described in Section 2.1.

The CRC+ dataset represented an increase in the approximate average number of non-annotated tiles per slide from 1075 to 1305. However, the approximate average number of tiles per annotated slide decreased from 2112 to 1264. This might represent a tougher task to solve on this dataset.

Two external datasets were also included for a domain generalisation evaluation. The first is composed by samples of the TCGA-COAD [30] and TCGA-READ [31] collections from The Cancer Imaging Archive [32], containing mostly surgical resection samples, excluding slides with pen markers, large air bubbles over tissue, tissue folds and other artefacts in large areas of the slide. We ended up with 232 samples reviewed and validated by the pathologist team, from which 230 of them were diagnosed as high-grade lesions, one as a low-grade lesion and one as non-neoplastic. The second external validation set is composed of 100 H&E slides from the Pathology AI Platform [33] colorectal cohort, which includes all the cases with more superficial sampling of the lesion, to better compare with our CRC+ dataset. All samples were also reviewed and validated as high-grade lesions by the pathologist team.

### 2.5. Training Details

We trained the convolutional neural network using Pytorch with the Adaptive Moment Estimation (Adam) optimiser, a learning rate of $6\times 10^{-6}$, a weight decay of $3\times 10^{-4}$ and a batch size of 32, for both the strongly and weakly supervised training steps. For the inference step in the weakly supervised approach, we used a batch size of 256 and the network was set to evaluation mode. The performance of the method was evaluated at the end of each epoch to select the best model based on the QWK. The training was conducted on a single Nvidia Tesla V100 (32 GB) GPU for 5 strongly supervised epochs and 30 weakly supervised epochs.

Seven feature vectors from the worst tiles were concatenated to train the aggregation methods. This led to a feature vector of size 3584. Afterwards, these feature vectors were used as input to train the aggregators developed with the help of the scikit-Learn library. In addition to this, the MLP aggregator required additional training parameters. It was optimised with stochastic gradient descent, mini-batches of 32 samples and an initial learning rate of $10^{-3}$.

## 3. Results

Due to the limited number of studies published on CRC diagnosis using DL methods and WSI, there are also a limited number of results comparable to ours. Thus, we decided to conduct our evaluation process in four different steps. First, we performed a quantitative evaluation of the aggregation methods and compared the results with Oliveira et al. [17], in Section 3.1. Then, we extended the dataset to a larger version with a total of 4433 samples, where we evaluated the top-performing aggregation methods and the baseline in Section 3.2. Afterwards, as in Section 4.1, we performed a qualitative assessment from an interpretability perspective: we retrieved a mapping of the tiles classification over the original image. Finally, we evaluated our method on two external datasets, as in Section 3.3.

### 3.1. Original CRC Dataset Evaluation

We evaluated our approach with the MIL-aggregation (max-pooling) and with eight different types of aggregators, as seen in Table 2. Approaches with tile aggregation at inference are, in general, better than the baseline method for CRC diagnosis from WSI. From those, the MLP aggregator using seven feature vectors outperformed the baseline and all the others aggregation schemes. Other approaches with aggregation showed overall good results, but are not on par with the MLP approach. In addition, the MLP has an increased specificity (by reducing the number of false positives) while avoiding a significant degradation of the sensitivity. The SVM and the K-NN have the best results from the remaining approaches, with the K-NN achieving the same specificity as MLP. Finally, the two voting approaches show notable improvements over the stand-alone aggregation methods, with the combination of SVM and KNN beating all the previous approaches on nearly every metric and achieving the same specificity of the MLP.

In Table 3 we can see the confusion matrix for the MLP (75;5), which was the best-performing method. It is worth noting that MLP (75;5) did not fail any prediction by more than one consecutive class (for instance, predicting HG as NNeo or vice-versa). This ensures that HG lesions are at least classified as LG or HG, which can be seen a desired feature of the model. When analysed as a binary classification problem, it is possible to observe that only 5 samples out of 259 are misclassified. This means that the proposed model shows a binary ACC of 98.1%.

**Figure 5:**
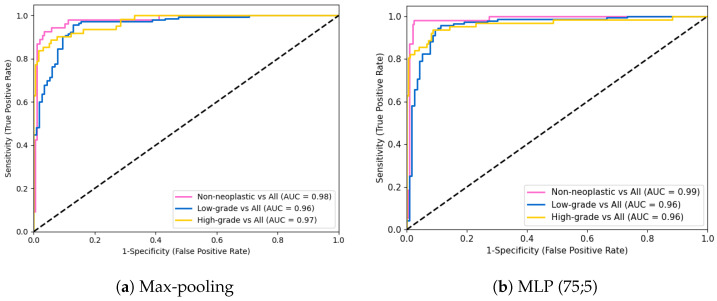
ROC curves for max-pooling and MLP (75;5) aggregator.

We also plotted the receiver operating characteristic (ROC) curve of the baseline and the best aggregation method. It was intended to verify not only their area under the curve (AUC), but the performance of the model per class. Once more, as seen in Figure 5, the MLP method outperformed the other approach in almost every class. Moreover, as expected, it is easier to distinguish non-neoplastic cases from the rest, than to decide between low- and high-grade lesions.

### 3.2. CRC+ Dataset Evaluation

We aimed to understand the relevance of adding additional annotated and non-annotated data to the performance of the algorithm. Hence, the results in Table 4 show the performance of the model with the original dataset, with increased annotated samples and with an increased number of non-annotated samples. In addition, we further introduced another version of the MLP aggregator, which comprises different layer dimensions, to test if the increased number of samples required more complex models. Surprisingly, the results did not evolve as expected, since the performance was negatively affected by the increase in the size of the dataset. This is likely caused by the overfitting of the aggregation method to the training data, which leads to a poor generalisation capability on test data.

In an attempt to fully understand the reason behind the performance drop, we created new training and test sets, with the latter being roughly 3.89 times larger than its previous version. The results of this new experiment are presented in the last three rows of Table 4. The improvements shown by training and evaluating on these larger training and test sets indicate that the smaller test set used for evaluation in Table 4 might have noisy labels or not be representative enough. Hence, the proposed model seems to be robust when given more training data and a larger test set. Finally, the superior performance of the aggregators on the new dataset split shows its relevance to the construction of well-balanced and accurate algorithms.

### 3.3. Domain Generalisation Evaluation

The development of medical-oriented deep neural networks is usually strongly influenced by the data source. Colour, saturation and image quality are important factors for the performance of these networks. Moreover, the type of sample is also important; for instance, despite the shared similarities, biopsies and surgical resection samples are quite distinct from each other. Hence, to evaluate the domain generalisation, the proposed method trained on CRC+ biopsies samples was evaluated on two external public datasets. The results are presented in Table 5 and Table 6.

As expected, due to its high sensitivity, and since almost all cases evaluated are high-grade cases, the max-pooling approach achieves the best results in terms of multiclass ACC, binary ACC and sensitivity. Regarding the TCGA dataset, these results can be explained by the fact that these samples are mostly from surgical resections, with bigger portions of tissue, whereas ours are from biopsies/polipectomies. Moreover, the datasets are somewhat different regarding the represented classes, with TCGA containing more poorly differentiated and mucinous adenocarcinomas, which are underrepresented in the CRC+ training set. Finally, the lower tissue image quality, when compared to the CRC+ dataset, may also explain this performance drop.

Regarding the better results on PAIP dataset, it can be explained by the better quality of the WSIs and an H&E staining colour being closer to CRC+ dataset. Moreover, although all PAIP slides seem to derive from surgical specimens, the sampling of the neoplasias was more superficial in most of the cases used (representing mostly mucosa and submucosa layers) as opposed to TCGA samples, in which many samples showcased all colonic layers (mucosa, submucosa, muscular and adipose tissue), differing greatly from the biopsies and polipectomies of the CRC+ dataset.

Domain generalisation is a complex topic that derives from several variables. In our scenario, the model displays a good capability to comprehend the content of a WSI collected on another lab, as seen in Table 6. However, there is still work to be done on the generalisation capability between strong colour differences and the capability of also assessing surgical specimens samples.

## 4. Discussion

### 4.1. Interpretability Assessment

**Figure 6:**
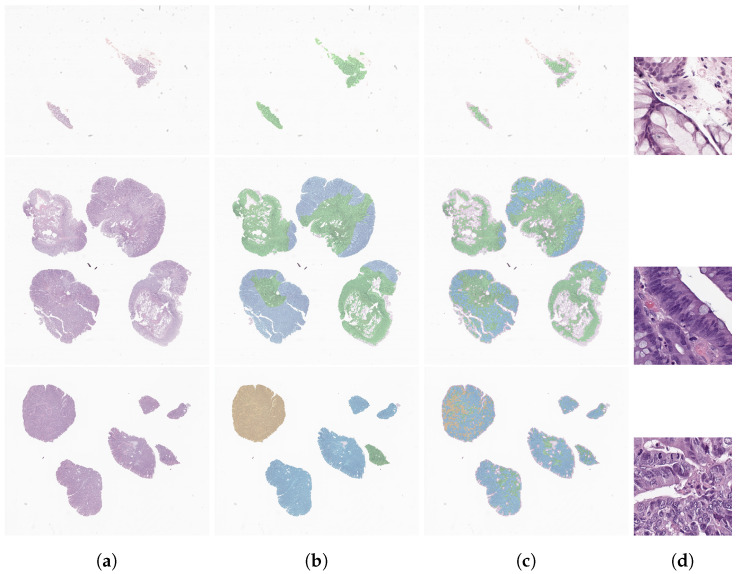
Examples of a model prediction map for each class, from the annotated data subset: a non-neoplastic case (**top**), a low-grade lesion (**middle**) and a high-grade lesion (**bottom**). Each column has the slides examples (**a**), the ground-truth annotation (**b**), the map with the tile predictions (**c**) and the most relevant tile (512×512 px), with the worst clinical class (**d**). The non-neoplastic, low-grade and high-grade regions are represented in green, blue and yellow, respectively.

**Figure 7:**
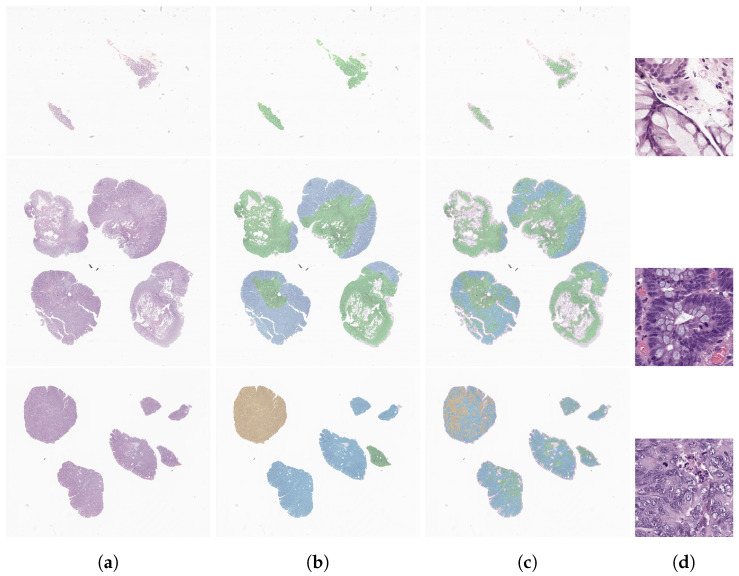
Examples of a model prediction map for each class, from the annotated data subset: a non-neoplastic case (**top**), a low-grade lesion (**middle**) and a high-grade lesion (**bottom**). Each column has the slides examples (**a**), the ground-truth annotation (**b**), the map with the tile predictions (**c**) and the most relevant tile (512×512 px), with the worst clinical class (**d**). The non-neoplastic, low-grade and high-grade regions are represented in green, blue and yellow, respectively.

In order to assess how the model classified each tile and to better understand the class distribution within each case, we retrieved the single tile predictions and assigned them to their respective position in the slide, creating a predictions map. For each case, we also retrieved the worst tile (in clinical terms). This experiment was conducted with slides from the annotated data subset (Figure 6) and further analysed by pathologists. By constructing these maps, we allowed pathologists to understand the reasoning of the model behind a slide prediction. Moreover, if necessary, it can guide and direct the focus of the pathologist to relevant areas in order to improve the overall workflow in clinical environments. As can be seen in the third column of Figure 6, although the model was not trained for segmentation, nor focused on individual tile-label prediction, the results are quite accurate in terms of lesion localisation, when compared to the ground truth (second column of Figure 6). On slides classified as NNeo and LG, the precision of the tile classification compared to the pathologists’ masks is rather impressive. For the HG slides, despite the lower density of tiles predicted as HG, the model was capable of capturing the majority of the fragments affected, as we verified on the maps generated for all the annotated slides. Moreover, for the same samples, we also retrieved the maps produced with the model trained on the full dataset (Figure 7). When compared with the previous examples, we can conclude that the model maintains the capability of locating tumour regions, especially in the high-grade example, without compromising the identification of non-neoplastic or low-grade areas.

## 5. Conclusions

The method proposed in this document presents significant improvements over state-of-the-art methods for colorectal cancer diagnosis. Not only are metrics such as ACC and the QWK better, but the sensitivity achieves values close to the maximum of 1. Furthermore, the method was trained and tested on an extended version of one of the largest datasets of colorectal histological samples publicly available, which increases the robustness of the test results and our trust in its metrics. Finally, the model was validated on external datasets for domain generalisation. Despite the performance drop in the TCGA dataset, when compared to CRC+ dataset, and some misclassifications in the PAIP dataset, it is worth noting that the model can detect high-grade lesions reasonably well, even in sets with many distinct properties compared to the one used for training.

Although achieving remarkable performance, medical applications of DL-based methods have been exposed to severe criticism due to its natural black-box structure. Here, we presented a model that attempts to support slide decision reasoning in terms of the spatial distribution of lesions. With this, we argue that our model is closer to being capable of being integrated into clinical practice to assist and ease the workload of pathologists. In addition to this interpretability analysis, we developed an aggregation system that is state-of-the-art in classifying CRC from digitised H&E slides. Further efforts should be devoted to bringing these methods closer to clinical applicability. From increased datasets, to better standardisation techniques, the future is bright. However, digital pathology is a young field, with much to improve over the following years.

## Figures and Tables

**Table 1:** CRC dataset summary, with the number of slides (annotated samples are detailed in parenthesis) and tiles distributed by class.

		NNeo	LG	HG	Total
	*# slides*	300 (6)	552 (35)	281 (59)	1133 (100)
CRC dataset [17]	*# annotated tiles*	49,640	77,946	83,649	211,235
	*# non-annotated tiles*	-	-	-	1,111,361
	*# slides*	663 (12)	2394 (207)	1376 (181)	4433 (400)
CRC+ dataset	*# annotated tiles*	145,898	196,116	163,603	505,617
	*# non-annotated tiles*	-	-	-	5,265,362

**Table 2:** Comparison of feature aggregation methods against the approach of Oliveira et al. [17], on the same test set. Both the ACC and the QWK score were computed for a three-classes problem, whereas the sensitivity and the specificity were computed for a binary problem by considering the LG and HG classes as an unique class. In bold are the best results per column.

Method	Annotated Samples	Training Tiles (L)	Aggregation Tiles $\left(L_{a}\right)$	QWK	ACC	Sensitivity	Specificity
Oliveira et al.	0	1	1	0.795	84.17%	0.933	-
Oliveira et al.	100	1	1	0.863	88.42%	0.957	-
Supervised baseline	100	-	1	0.027	29.73%	0.449	0.796
Max-pooling	100	5	1	0.881	91.12%	**0.990**	0.852
MLP (75;5)	100	5	7	**0.906**	**91.89%**	0.980	**0.981**
SVM				0.887	90.35%	0.971	0.944
KNN				0.890	90.35%	0.971	**0.981**
RF				0.878	89.57%	0.966	0.963
AdaBoost				0.862	88.03%	0.961	0.907
XGBoost				0.879	89.58%	0.961	0.963
SVM + KNN	100	5	7	0.898	91.12%	0.971	**0.981**
SVM + RF + KNN	100	5	7	0.893	90.73%	0.971	**0.981**

**Table 3:** Confusion matrix of the MLP (75;5) in the multiclass setup, using the CRC test set (259 samples), with non-neoplastic (NNeo), low-grade (LG) and high-grade (HG) classes. In bold are the best results per column.

		Predicted		
**Actual**		*NNeo*	*LG*	*HG*
	*NNeo*	**53**	1	0
	*LG*	4	**137**	2
	*HG*	0	14	**48**

**Table 4:** Model performance evaluation with increasing training sets and/or annotated samples (in parenthesis). Both the ACC and the QWK score were computed for a three-classes problem, whereas the sensitivity and the specificity were computed for a binary classification problem by considering the LG and HG classes as one unique class. In bold are the best results per column.

Method	Training Samples	Test Samples	Aggregation Tiles $\left(L_{a}\right)$	QWK Score	ACC	Sensitivity	Specificity
Max-pooling	874 (100)	259	1	0.881	91.12%	**0.990**	0.852
MLP (75;5)			7	**0.906**	**91.89%**	0.980	**0.981**
MLP (300;50)			7	0.885	91.12%	0.966	**0.981**
Max-pooling	1174 (400)	259	1	**0.874**	**91.12%**	**0.985**	0.907
MLP (75;5)			7	0.838	86.49%	0.946	0.926
MLP (300;50)			7	0.850	87.26%	0.941	**0.944**
Max-pooling	4174 (400)	259	1	**0.834**	**89.96%**	**0.980**	0.870
MLP (75;5)			7	0.810	83.78%	0.922	0.889
MLP (300;50)			7	0.816	83.01%	0.927	**0.926**
Max-pooling	3424 (400)	1009	1	0.884	89.89%	**0.992**	0.815
MLP (75;5)			7	0.871	88.89%	0.982	0.839
MLP (300;50)			7	**0.888**	**90.19%**	0.988	**0.857**

**Table 5:** Model performance evaluation on the TCGA test set. In bold are the best results per column.

Method	ACC	Binary ACC	Sensitivity
Max-pooling	**71.55%**	**80.60%**	**0.805**
MLP (75;5)	61.20%	75.43%	0.753
MLP (300;50)	58.62%	74.13%	0.740

**Table 6:** Model performance evaluation on the PAIP test set. In bold are the best results per column.

Method	ACC	Binary ACC	Sensitivity
Max-pooling	**99.00%**	**100.00%**	**1.000**
MLP (75;5)	77.00%	98.00%	0.980
MLP (300;50)	77.00%	98.00%	0.980

## Data Availability

The CRC and CRC+ datasets, from IMP Diagnostics, are available on reasonable request through the following email contact: cadpath.ai@impdiagnostics.com. Data used for validation is available from the TCGA Research Network (https://cancergenome.nih.gov/ last accessed on (20 April 2022)) and from the Pathology AI Platform, PAIP (http://www.wisepaip.org/paip last accessed on (20 April 2022)).

## Abbreviations

The following abbreviations are used in this manuscript:

ACC	Accuracy
AD	Adenoma
Adam	Adaptive moment estimation
ADC	Adenocarcinoma
AI	Artificial intelligence
AUC	Area under the (ROC) curve
CNN	Convolutional neural network
CRC	Colorectal cancer
DL	Deep learning
ESGE	European Society of Gastrointestinal Endoscopy
GPU	Graphics processing unit
H&E	Haemotoxylin and eosin
HGD	High-grade dysplasia
K-NN	K-nearest neighbour
LGD	Low-grade dysplasia
MIL	Multiple instance learning
MLP	Multilayer perceptron
NNeo	Non-neoplastic
QWK	Quadratic weighted kappa
RF	Random forest
RNN	Recurrent neural network
ROC	Receiver operating characteristic
SVM	Support vector machine
WSI	Whole slide image(s)
